# Integrally Cooperative Spatio-Temporal Feature Representation of Motion Joints for Action Recognition

**DOI:** 10.3390/s20185180

**Published:** 2020-09-11

**Authors:** Xin Chao, Zhenjie Hou, Jiuzhen Liang, Tianjin Yang

**Affiliations:** School of Computer Science and Artificial Intelligence, Changzhou University, Changzhou 213164, China; chaoxin941203@163.com (X.C.); jzliang@cczu.edu.cn (J.L.); yangtianjin128@163.com (T.Y.)

**Keywords:** human action recognition, Motion Collaborative Spatio-Temporal Vector, Motion Spatio-Temporal Map, Multi-Target Subspace Learning, key information extraction based on inter-frame energy fluctuation

## Abstract

In contemporary research on human action recognition, most methods separately consider the movement features of each joint. However, they ignore that human action is a result of integrally cooperative movement of each joint. Regarding the problem, this paper proposes an action feature representation, called Motion Collaborative Spatio-Temporal Vector (MCSTV) and Motion Spatio-Temporal Map (MSTM). MCSTV comprehensively considers the integral and cooperative between the motion joints. MCSTV weighted accumulates limbs’ motion vector to form a new vector to account for the movement features of human action. To describe the action more comprehensively and accurately, we extract key motion energy by key information extraction based on inter-frame energy fluctuation, project the energy to three orthogonal axes and stitch them in temporal series to construct the MSTM. To combine the advantages of MSTM and MCSTV, we propose Multi-Target Subspace Learning (MTSL). MTSL projects MSTM and MCSTV into a common subspace and makes them complement each other. The results on MSR-Action3D and UTD-MHAD show that our method has higher recognition accuracy than most existing human action recognition algorithms.

## 1. Introduction

Human action recognition [1] is a research hotspot in the field of artificial intelligence and pattern recognition. The research achievements have been used in many aspects of life [2], such as human-computer interaction, biometrics, health monitoring, video surveillance systems, somatosensory game, robotics, etc. [3].

Due to the development of lower-cost depth sensors, deep cameras have been widely used in action recognition. Compared with traditional red, green and blue (RGB) cameras, the depth camera is not sensitive to lighting conditions [4]. It is easy to distinguish the background and foreground, and provides human depth data. In addition, human skeletal information can also be obtained from the depth map.

So far, many scholars have used depth sequences for human action recognition research. Li et al. [5] selected representative 3D points to depict human action. Oreifej et al. [6] proposed histogram of oriented 4d normals (HON4D) to capture the structural information of action. Yang et al. [7] proposed depth motion map (DMM) to accumulate the motion energy of neighboring moments.

Since the skeletal information can directly describe the movement of human joints, many scholars have begun to use skeleton sequences to recognize human action. Xia et al. [8] proposed histograms of 3D joints (HOJ3D) as a compact representation of human action. Vemulapalli et al. [9] represented human actions as curves that contain skeletal action sequences. They modeled the 3D geometric correlations among different body parts by using rotations and translations. Luvizon et al. [10] proposed a robust method based on vector of locally aggregated descriptors (VLAD) algorithm and clustering library, which extracts spatiotemporal local feature sets from joint subgroups to represent human action.

In the field of action recognition, the use of single modal data is one-sided, and it is necessary to integrate different modals data for comprehensive decision-making. Many fusion methods have been successfully applied to action recognition, among which feature fusion is most extensive. Canonical Correlation Analysis (CCA) [11] and Discriminant Correlation Analysis (DCA) [12] are commonly used feature fusion methods.

**Figure 1:**
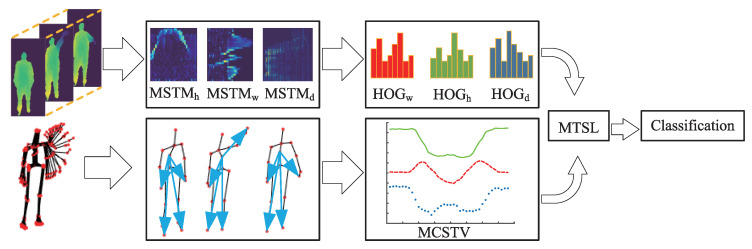
Workflow illustration of our method.

Although significant progress has been made in human action recognition, there are still shortcomings. Currently, many studies only independently perform feature extraction [13] and recognition of some motive joints. However, they did not consider the comprehensiveness, integration and collaboration between the joints. For example, in throwing, the main movement part of the body is right hand. If we only consider the movement features of right hand, this action is likely to be recognized as a waving. If we combine the movements of the left hand, left and right legs of the body with those of the right hand, then the possibility of this action being recognized as a throwing is greatly enhanced. Regarding the problem, this paper proposes an action feature representation algorithm that considers the integrally cooperative movement features of human action, called Motion Collaborative Spatio-Temporal Vector (MCSTV) and Motion Spatio-Temporal Map (MSTM). MCSTV reflects the integrally cooperative of human action through weighted accumulating limbs’ motion vector. MSTM can accurately describe the spatial structure [14] and temporal information [15] of actions, we extract key motion energy by key information extraction based on inter-frame energy fluctuation, and project the key energy of body on three orthogonal axes and stitched according to temporal series to form three-axis MSTMs. To give full play to the advantages of MSTM and MCSTV, we propose Multi-Target Subspace Learning (MTSL). MTSL projects MSTM and MCSTV into a common subspace, alienates the inter-class distance of samples of different categories and reduces the dimension of projection target area by constructing multiple projection target centers of each sample of the same category. The workflow illustration of our method is shown in Figure 1.

In this paper, we focus on the challenging problem of action recognition. Our contributions are as follows:

- Motion Collaborative Spatio-Temporal Vector is proposed, which is a feature representation method that comprehensively considers the integral and cooperative of human action.
- Motion Spatio-Temporal Map is proposed, which is a feature representation method that fully preserves the spatial structure and temporal information of human action.
- Key information extraction based on inter-frame energy fluctuation is proposed, which is a method that extracts key information in motion energy.
- Multi-Target Subspace Learning is proposed, which is used to fuse MCSTV and MSTM.

This paper is organized as follows. In Section 2, the related work is briefly reviewed. In Section 3, method is detailly described. The results of experimental and discussions are presented in Section 4. Finally, Section 5 summarizes the paper.

## 2. Related Work

### 2.1. Action Recognition Based on Skeleton Approach

Human action recognition based on skeleton approach is a research hotspot. Lv et al. [16] decomposed the high dimensional 3D joint space into a set of feature space. Then, the hidden Markov model (HMM) combined with multi-class AdaBoost (Adabbs.m2) algorithm and dynamic programming algorithm are used to segment and recognize the actions. Hussein et al. [17] presented an action recognition algorithm from 3D skeleton sequences extracted from depth data. This method uses the covariance matrix for skeleton joint locations over time as a discriminative descriptor for a sequence. Zanfir et al. [18] proposed a non-parametric Moving Pose (MP) framework for human action recognition. They captured both pose information and the speed and acceleration of human joints and finally used a modified K-Nearest Neighbor (KNN) classifier in conjunction with the descriptors to classify human actions. However, these methods did not consider the comprehensiveness, integration and collaboration between the joints.

### 2.2. Motion Energy Image and Motion History Image

In the early stage of human action recognition, various methods were used to extract features from color video collected by RGB cameras to complete recognition. Bobick et al. [19] proposed Motion Energy Image (MEI) and Motion History Image (MHI). The MEI algorithm needs to extract the foreground area of movement. Then the foreground region is binarized to obtain the binary image sequence of the action. Finally, the union of binary image sequences is taken to obtain MEI of the action. The calculation of MEI is expressed as:

(1) $${\mathrm{MEI}}_{\delta }\left(x,y,t\right)=\overset{\delta -1}{\underset{i=0}{⋃}}B\left(x,y,t-i\right)$$

where ${\mathrm{MEI}}_{\delta }\left(x,y,t\right)$ represents that MEI is generated by δ images at the *t*-th frame in the video sequence. B(x,y,t) represents the *t*-th frame of the binary image sequence. *x* and *y* respectively represent the height and width of a pixel point on the image. *t* represents the sequence number of a frame in the video sequence.

The MEI describes the largest contour of actions. It loses some motion information inside the contour and cannot express the temporal information of actions.

Unlike MEI, MHI is a grayscale image. The pixel intensity is a function of the temporal history of motion at that point. A simple replacement and decay operator can be used:

(2) $$\begin{array}{c} {\mathrm{MHI}}_{\tau }\left(x,y,t\right)=\left\{\begin{array}{cc} \tau & \;\mathrm{if}\;B(x,y,t)=1\\ \max\left(0,{\mathrm{MHI}}_{\tau }\left(x,y,t-1\right)-1\right) & \;\mathrm{otherwise}\; \end{array}\right. \end{array}$$

where τ is the initial brightness; ${\mathrm{MHI}}_{\tau }\left(x,y,t\right)$ is the MHI generated by the *t* frames of the video sequence.

Compared with MEI, MHI retains part temporal information of actions through brightness attenuation. However, MHI also cannot fully express the spatial structure.

### 2.3. Action Recognition Based on Depth Approach

With the development of depth sensors, scholars began to use depth sequence to study human action recognition. Numerous scholars [20] use depth motion map (DMM) for action recognition research. Each frame in the depth sequence is projected onto three orthogonal cartesian planes to form three 2D projection maps according to front, side, and top views, denoted ${\mathrm{map}}_{f}$, ${\mathrm{map}}_{s}$, ${\mathrm{map}}_{t}$. The motion energy of two consecutive projection images is respectively accumulated to form the DMM of three views. The calculation of DMM is expressed as:

(3) $${\mathrm{DMM}}_{v}=\sum_{i=1}^{N}\left(\left|{\mathrm{map}}_{v}^{i+1}-{\mathrm{map}}_{v}^{i}\right|>\varepsilon \right)$$

where v∈{f,s,t} represents the projection view, f is front view, s is side view, t is top view. ${\mathrm{DMM}}_{v}$ is the DMM of view *v*. ${\mathrm{map}}_{v}^{i+1}-{\mathrm{map}}_{v}^{i}$ denotes the image difference between the *i*-th frame and the i+1-th frame, namely the motion energy between the *i*-th frame and the i+1-th frame. *N* represents the number of frames of the depth sequence. ε indicates a difference threshold.

Compared with MEI, DMM fully reflects the depth information of actions. However, DMM also cannot express the temporal information.

Yang et al. [7] computed Histograms of Oriented Gradients (HOG) from DMM as the representation of an action sequence. Chen et al. [20] extracted Local Binary Pattern (LBP) from DMM. Chen et al. [21] extracted Gradient Local Auto-Correlations (GLAC) from DMM. Zhang et al. [22] presented 3D histograms of texture (3DHoTs) to extract discriminant features from depth video sequences. Wang et al. [23] extracted random occupancy pattern (ROP) features from depth video sequences and use a sparse coding approach to encode these features. Xia et al. [24] built depth cuboid similarity features (DCSF) around the local spatio-temporal interest points (STIPs) extracted from depth video sequences to describe local 3D depth cuboids. Vieira et al. [25] presented Space-Time Occupancy Patterns (STOP) to preserve spatial and temporal information between space-time cells.

### 2.4. Feature Fusion

Feature fusion can make single features complement each other to improve recognition accuracy. Many achievements have been made in this field. For example, Hardoon et al. [11] proposed Canonical Correlation Analysis (CCA) which maximizes the correlation between two different features. Zhang et al. [26] proposed group sparse canonical correlation analysis to preserve the group sparse characteristics of data within each set in addition to maximize the global covariance. Haghighat et al. [12] proposed DCA that performs feature fusion by maximizing the pairwise correlations across the two feature sets and eliminating the inter-class correlations and restricting the correlations to be in the classes. Wang et al. [27] proposed Joint Feature Selection and Subspace Learning (JFSSL) for for cross-modal retrieval, in which multimodal features are projected into a common subspace by learning projection matrices. However, these methods generally describe the correlation between different features and do not consider the correlation between the samples of different categories.

## 3. Method

As shown in Figure 1, we extract MSCTV from skeleton sequences, and extract MSTM from depth video sequences. Next, we further extract the Histogram of Oriented Gridient (HOG) feature of MSTM. Then, we fuse MCSTV and MSTM through MTSL, and use the fusion features to complete human action recognition research.

### 3.1. Motion Collaborative Spatio-Temporal Vector

Human action is the fact of integrally cooperative movement of all joints, not the fact of individual movement of some joints. Therefore, this paper proposes Motion Collaborative Spatio-Temporal Vector that considers the integrally cooperative movement of limbs’ joints.

**Figure 2:**
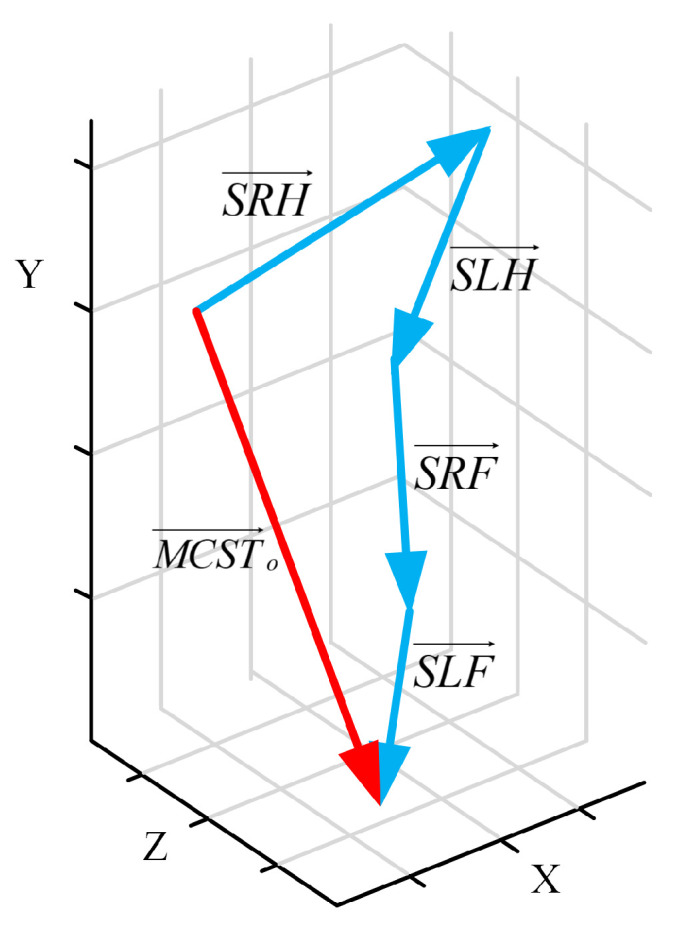
The basic idea of MCSTV (Contribution degree is not considered).

**Figure 3:**
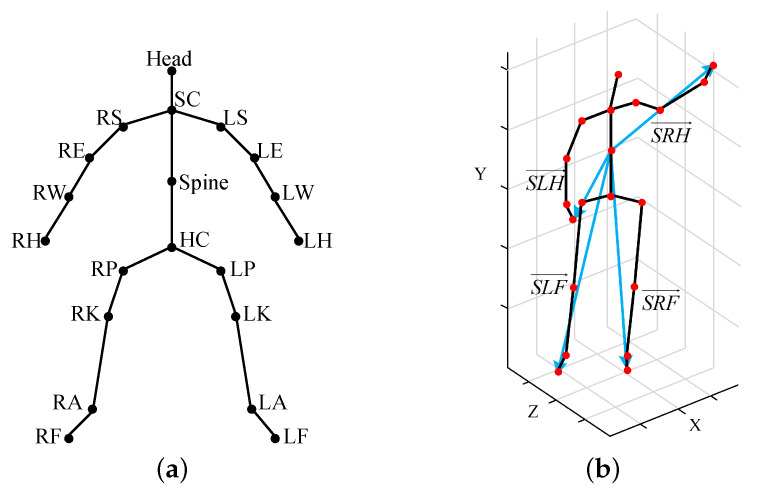
Human skeleton. (**a**) Skeleton; (**b**) The skeletal frame of the high wave.

Most actions are the result of multiple joints moving integrally. In this paper, the scattered and separate motion joints’ information is spliced by vector superposition to form a comprehensive vector that can comprehensively describe action and highlight the integral and cooperative of movement. The comprehensive vector is called MCSTV, the basic principle of MCSTV is shown in Figure 2. Where $\vec{SLH}$ represents the motion vector of left upper limb, $\vec{SRH}$ represents the motion vector of right upper limb, $\vec{SLF}$ represents the motion vector of left lower limb, $\vec{SRF}$ represents the motion vector of right lower limb, and ${\vec{MCST}}_{o}=\vec{SLH}+\vec{SRH}+\vec{SLF}+\vec{SRF}$, ${\vec{MCST}}_{o}$ represents the original MCSTV. Due to MCSTV is stitched by the limbs’ motion vector, MCSTV can reflect the fact of the comprehensive effect of multiple motion vectors to a certain degree. The human skeleton is shown in Figure 3a. The skeletal frame of the high wave is shown in Figure 3b.

As shown in Figure 3b, we select the motion vector from the spine to the left hand to represent the motion of left upper limb, that from the spine to the right hand to represent the motion of right upper limb, that from the spine to the left foot to represent the motion of left lower limb, and that from the spine to the right foot to represent the motion of right lower limb, respectively, denoted $\vec{SLH}$, $\vec{SRH}$, $\vec{SLF}$, $\vec{SRF}$.

For different actions, the contribution degree of each joint is different. As showed in Figure 2, the motion vector of the limbs are directly accumulated. Due to the degree of limb is different, if limbs’ motion vector are directly accumulated, the action cannot be accurately described. We must obtain the contribution degree of each limb. However, these motion vectors are all three-dimensional vectors, and the change of the vector in space is the result of the change of the vector in multi-view. Therefore, these motion vectors need to be projected onto three orthogonal cartesian planes xoy, yoz and xoz. The offset of $\vec{SLH}$ on each plane is expressed as:

(4) $$\begin{array}{c} \theta_{SLH_{xy}}\left(i\right)=\arccos\left(\frac{{\vec{SLH}}_{xy}\left(i+1\right)\times {\vec{SLH}}_{xy}\left(i\right)}{\left|{\vec{SLH}}_{xy}\left(i+1\right)\right|\left|{\vec{SLH}}_{xy}\left(i\right)\right|}\right)\\ \theta_{SLH_{yz}}\left(i\right)=\arccos\left(\frac{{\vec{SLH}}_{yz}\left(i+1\right)\times {\vec{SLH}}_{yz}\left(i\right)}{\left|{\vec{SLH}}_{yz}\left(i+1\right)\right|\left|{\vec{SLH}}_{yz}\left(i\right)\right|}\right)\\ \theta_{SLH_{xz}}\left(i\right)=\arccos\left(\frac{{\vec{SLH}}_{xz}\left(i+1\right)\times {\vec{SLH}}_{xz}\left(i\right)}{\left|{\vec{SLH}}_{xz}\left(i+1\right)\right|\left|{\vec{SLH}}_{xz}\left(i\right)\right|}\right) \end{array}$$

where ${\vec{SLH}}_{xy}\left(i\right)$, ${\vec{SLH}}_{yz}\left(i\right)$ and ${\vec{SLH}}_{xz}\left(i\right)$ respectively represent the projection of the *i*-th frame of $\vec{SLH}$ on xoy, yoz and xoz. $\theta_{SLH_{xy}}\left(i\right)$, $\theta_{SLH_{yz}}\left(i\right)$ and $\theta_{SLH_{xz}}\left(i\right)$ respectively represent the offsets of the *i*-th frame of $\vec{SLH}$ on xoy, yoz and xoz.

The offset of $\vec{SLH}$ of each frame is the sum of offset on three orthogonal planes. The offset of each frame of $\vec{SLH}$ is expressed as:

(5) $$\theta_{SLH}\left(i\right)=\theta_{SLH_{xy}}\left(i\right)+\theta_{SLH_{yz}}\left(i\right)+\theta_{SLH_{xz}}\left(i\right)$$

where $\theta_{SLH}\left(i\right)$ represents the offsets of the *i*-th frame of $\vec{SLH}$.

Each action consists of *N* frames, so the total offset of $\vec{SLH}$ is the sum of the offset of each frame. The offset of $\vec{SLH}$ is expressed as:

(6) $$sum_{SLH}=\sum_{i=1}^{N}\theta_{SLH}\left(i\right)$$

where $sum_{SLH}$ represents the total offset of $\vec{SLH}$.

Similarly, according to Equations (4)–(6), the total offset of $\vec{SRH}$ is obtained as $sum_{SRH}$, the total offset of $\vec{SLF}$ as $sum_{SLF}$ and the total offset of $\vec{SRH}$ as $sum_{SRF}$.

The contribution degree of each limb is the ratio of the offset of the limb and the total offset of all limbs. The contribution degree of each limb is expressed as:

(7) $$\begin{array}{c} W_{SLH}=\frac{sum_{SLH}}{sum_{SLH}+sum_{SRH}+sum_{SLF}+sum_{SRF}}\\ W_{SRH}=\frac{sum_{SRH}}{sum_{SLH}+sum_{SRH}+sum_{SLF}+sum_{SRF}}\\ W_{SLF}=\frac{sum_{SLF}}{sum_{SLH}+sum_{SRH}+sum_{SLF}+sum_{SRF}}\\ W_{SRF}=\frac{sum_{SRF}}{sum_{SLH}+sum_{SRH}+sum_{SLF}+sum_{SRF}} \end{array}$$

where $W_{SLH}$, $W_{SRH}$, $W_{SLF}$ and $W_{SRF}$ respectively represent the contribution degree of $\vec{SLH}$, $\vec{SRH}$, $\vec{SLF}$ and $\vec{SRF}$.

Finally, the contribution degree of each limb was used to constrain motion vector of each frame, and motion vector of each limb were weighted accumulated to form MCSTV. The calculation of MCSTV is expressed as:

(8) $$\begin{array}{c} {\vec{MCST}}_{w}\left(i\right)=\vec{SLH}\left(i\right)\times W_{SLH}+\vec{SRH}\left(i\right)\times W_{SRH}+\vec{SLF}\left(i\right)\times W_{SLF}+\vec{SRH}\left(i\right)\times W_{SRH} \end{array}$$

where ${\vec{MCST}}_{w}\left(i\right)$ represents the *i*-th frame of ${\vec{MCST}}_{w}$, ${\vec{MCST}}_{w}$ represents the MCSTV that motion vector of each limb were weighted accumulated.

**Figure 4:**
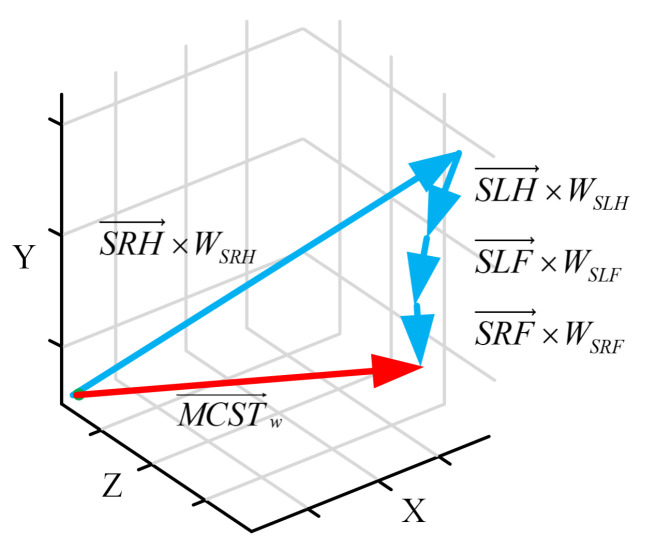
The final MCSTV.

MCSTV is obtained by weighted accumulation of motion vector of each limb is shown in Figure 4. As can be seen from the figure, after weighted accumulation, the MCSTV of this action is dominated by $\vec{SRH}$. Compared with the direct accumulation of motion vector in Figure 2, this method more directly reflects the major motion joints of the action.

### 3.2. Motion Spatio-Temporal Map

**Figure 5:**
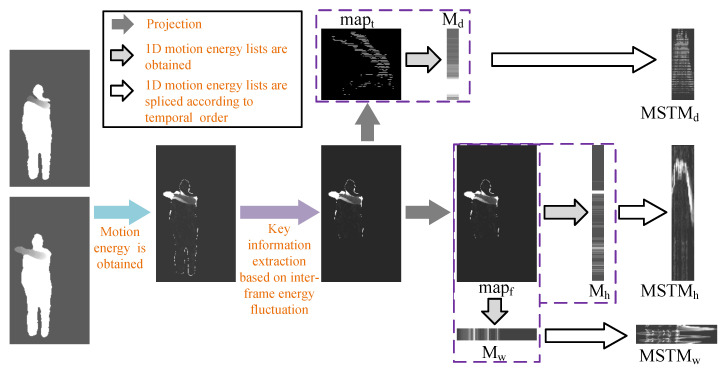
The flow illustration of MSTM.

To describe the action information more comprehensively and accurately, this paper proposes a feature representation algorithm, called Motion Spatio-Temporal Map (MSTM). MSTM can completely express the spatial structure and temporal information. This algorithm calculates the difference between adjacent frames of depth sequence to obtain the motion energy. Next, the key information is extracted from the motion energy by key information extraction based on inter-frame energy fluctuation. Then, the key energy is projected to three orthogonal axes to obtain the motion energy list of the three orthogonal axes. Finally, the motion energy list is spliced in temporal series to form MSTM. The flow illustration of MSTM is shown in Figure 5.

As shown in Figure 5, the motion energy of the action is obtained through the difference operation between two adjacent frames of the depth sequence. The motion energy is expressed as:

(9) $$E_{k}\left(x,y,z\right)=\left|I_{k+1}\left(x,y,z\right)-I_{k}\left(x,y,z\right)\right|$$

where $I_{k}\left(x,y,z\right)$ and $I_{k+1}\left(x,y,z\right)$ respectively represent the body energy of the *k*-th and *k*+1-th moment, i.e., the *k*-th and *k*+1-th frame of depth video sequence. $E_{k}\left(x,y,z\right)$ represents the motion energy of the *k*-th moment.

**Figure 6:**
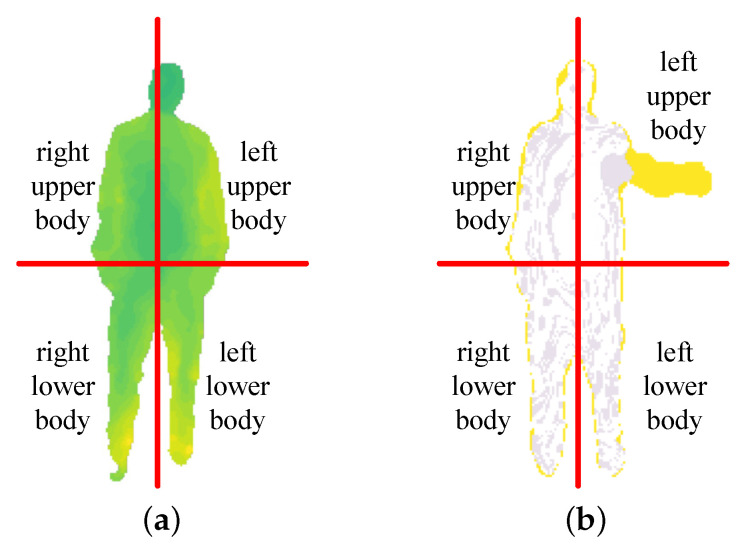
The division of human activity areas. (**a**) The division of human activity areas at the beginning of the action; (**b**) The division of human activity areas when the wave.

Due to the habitual sloshing of some joints, there is a lot of redundancy in the motion energy obtained by Equation (9). Regarding the problem, we propose an energy selection algorithm, i.e., key information extraction based on inter-frame energy fluctuation. We use this algorithm to remove the redundant of the motion energy at each moment. The main idea of this algorithm is to divide the human body into four areas according to the height range and width range at the initial moment of actions. Then, we calculate the proportion of the motion energy of each region in the whole body, and select a certain amount of motion energy as the main energy. The remaining energy is taken as redundancy and removed. The detailed steps of this algorithm are as follows:

Firstly, we calculate the height range $\left[h_{\min},h_{\max}\right]$ and width range $\left[w_{\min},w_{\max}\right]$ of human activity area at the initial moment of actions. The body is divided into upper body and lower body according to the hip center of the body. The body is divided into left body and right body according to the symmetry. Finally, the body is divided into four regions: left upper body (LU), right upper body (RU), left lower body (LL), and right lower body (RL). The motion energy of the body is the sum of the motion energy of four regions. The division of human activity areas is shown in Figure 6.

Next, we calculate the motion energy proportion of each region in the whole body, the energy proportion can be expressed as:

(10) $$\eta_{\psi }=\frac{\sum_{x=1}^{H_{\psi }}\sum_{y=1}^{W_{\psi }}E_{k}\left(x,y,z\right)}{\sum_{x=1}^{H}\sum_{y=1}^{W}E_{k}\left(x,y,z\right)}$$

where ψ∈{LU,RU,LL,RL}, $\eta_{\psi }$ represent the motion energy proportion of each region. *H*, *W* respectively represent the height and width of the whole body. $H_{\psi }$, $W_{\psi }$ respectively represent the height and width of each region.

Then, we rank the motion energy proportions of each region from the largest to the smallest. The maximum value is denoted as $\eta_{1}$, the minimum value is denoted as $\eta_{4}$, and $\eta_{1}>\eta_{2}>\eta_{3}>\eta_{4}$. In this paper, we select ξ of the whole body energy as the key energy. The remaining energy is considered to be redundant and removed from the original motion energy. The value of ξ is determined by the experimental results and recognition accuracies in Section 4.2.1. The selection of key energy follows the following criteria.

If $\eta_{1}>\xi$, then the motion energy of the corresponding region of $\eta_{1}$ is retained as the key energy, and the motion energy of the other three regions is considered to be redundant. If $\eta_{1}<\xi$ and $\eta_{1}+\eta_{2}>\xi$, then the motion energy of the corresponding region of $\eta_{1}$ and $\eta_{2}$ is retained as the key energy, and the motion energy of the other two regions is considered to be redundant. If $\eta_{1}+\eta_{2}<\xi$ and $\eta_{1}+\eta_{2}+\eta_{3}>\xi$, then the motion energy of the corresponding region of $\eta_{1}$, $\eta_{2}$ and $\eta_{3}$ is retained as the key energy, and the motion energy of $\eta_{4}$ is considered to be redundant. If none of the above conditions are met, the whole body motion energy is considered to be the key energy and retained.

The key energy is projected onto three orthogonal cartesian planes to form three 2D projection maps according to front view, side view, and top view, denoted ${\mathrm{map}}_{f}$, ${\mathrm{map}}_{s}$, ${\mathrm{map}}_{t}$. The 2D projection maps are expressed as:

(11) $$\begin{array}{c} {\mathrm{map}}_{f}\left(x,y\right)=E\left(x,y\right)\\ {\mathrm{map}}_{s}\left(x,z\right)=E\left(x,z\right)\\ {\mathrm{map}}_{t}\left(y,z\right)=E\left(y,z\right) \end{array}$$

where ${\mathrm{map}}_{f}\left(x,y\right)$, ${\mathrm{map}}_{s}\left(x,z\right)$ and ${\mathrm{map}}_{t}\left(y,z\right)$ respectively represent the coordinate of a pixel point on the ${\mathrm{map}}_{f}$, ${\mathrm{map}}_{s}$ and ${\mathrm{map}}_{t}$. E(x,y), E(x,z), and E(y,z) respectively represent the value of a pixel point on the ${\mathrm{map}}_{f}$, ${\mathrm{map}}_{s}$ and ${\mathrm{map}}_{t}$.

To obtain the energy distribution of the width axis, height axis and depth axis, we select ${\mathrm{map}}_{f}$ and ${\mathrm{map}}_{t}$ to continue to project to the corresponding orthogonal axis to obtain the row sum or column sum of the 2D energy projection maps. According to width axis, height axis and depth axis, three 1D motion energy lists are generated, which are expressed as $M_{w}$, $M_{h}$ and $M_{d}$ respectively. The 1D motion energy list is expressed as:

(12) $$\begin{array}{c} M_{w}\left(j\right)=\sum_{x=1}^{H_{m}}{\mathrm{map}}_{f}\left(x,j\right)\\ M_{h}\left(j\right)=\sum_{y=1}^{W_{m}}{\mathrm{map}}_{f}\left(j,y\right)\\ M_{d}\left(j\right)=\sum_{y=1}^{W_{m}}{\mathrm{map}}_{t}\left(j,y\right) \end{array}$$

where $M_{w}\left(j\right)$, $M_{h}\left(j\right)$, and $M_{d}\left(j\right)$ respectively represent the *j*-th element of the energy list on the width axis, height axis, and depth axis. $W_{m}$ and $H_{m}$ respectively represent the width and height of the 2D energy projection map.

According to the temporal order, $M_{u}$ are spliced to form MSTM of three axes, which are respectively represented as ${\mathrm{MSTM}}_{w}$, ${\mathrm{MSTM}}_{h}$ and ${\mathrm{MSTM}}_{d}$. For the depth sequence of *N* frames, the calculation of MSTM is expressed as:

(13) $${\mathrm{MSTM}}_{u}\left(k\right)=M_{u}^{k}$$

where u∈{w,h,d}, w is width axis, h is height axis and d is depth axis, $M_{u}^{k}$ represents the 1D motion energy list of the *k*-th frame of the action sequence on the *u* axis. ${\mathrm{MSTM}}_{u}$ represents the MSTM on the *u* axis. ${\mathrm{MSTM}}_{u}\left(k\right)$ represents the *k*-th row of ${\mathrm{MSTM}}_{u}$.

With the maximum width $W_{\max}$, minimum width $W_{\min}$, maximum height $H_{\max}$ and minimum height $H_{\max}$ of the body’s activity area as the bounds, MSTM is processed with the region of interest (ROI) [28], i.e., the image is cropped and normalized.

**Figure 7:**
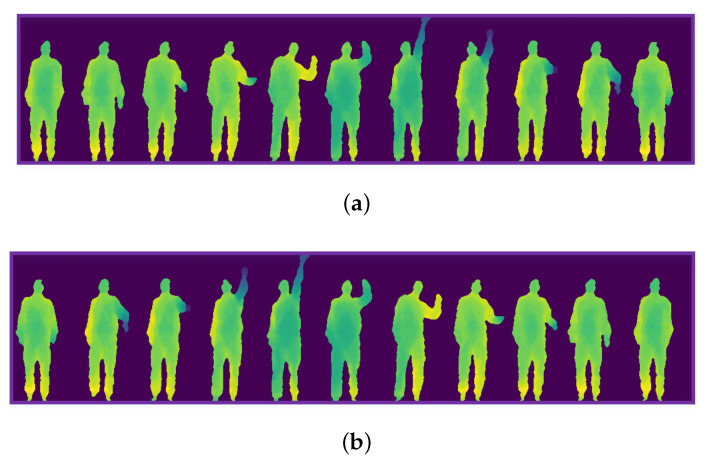
High throw. (**a**) The positive order of high throw; (**b**) The reverse order of high throw.

**Figure 8:**
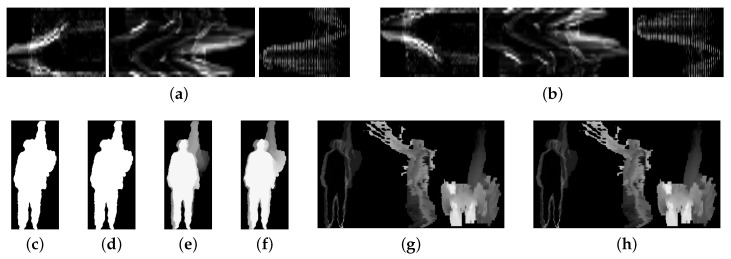
The various feature maps of the positive and reverse order high throw. (**a**) MSTM of positive order high throw; (**b**) MSTM of reverse order high throw; (**c**) MEI of positive order high throw; (**d**) MEI of reverse order high throw; (**e**) MHI of positive order high throw; (**f**) MHI of reverse order high throw; (**g**) DMM of positive order high throw; (**h**) DMM of reverse order high throw.

The actions in the original depth sequence are defined as positive order actions. The positive order high throw is shown in Figure 7a. The actions that the order is contrary to the original depth sequence are defined as reverse order actions. The reverse order high throw is shown in Figure 7b. The various feature maps of the positive and reverse order high throw are shown in Figure 8.

Figure 8a,b are the MSTM of positive order and reverse order high throw respectively. From left to right are the MSTM of height axis, width axis and depth axis respectively. Owing to MSTM reflects the change of the energy information on the three orthogonal axes, it preserves the spatial and temporal information completely. Positive order and reverse order actions have the same motion trajectories and opposite temporal orders, the final MSTM is symmetric along the time axis and easy to be distinguish. In contrast, Figure 8e,f respectively represent the MHI of positive order and reverse order high throw. MHI retains part of temporal information of the actions and has the ability to distinguish between positive order and reverse order actions. However, due to the coverage of the trajectory and the absence of the depth information, MHI cannot fully express the spatial information. Figure 8c,d are the MEI of positive order and reverse order high throw, respectively. Figure 8g,h are the DMM of positive order and reverse order high throw, respectively. MEI and DMM do not involve temporal information, so they cannot be distinguish. MEI does not involve the depth information, which means the spatial information is incomplete. DMM contains depth information and expresses spatial information fully.

### 3.3. Feature Fusion

To ensure that the description of action information more accurate, fuse features are usually used in human action recognition research. Therefore, this paper fuses skeleton feature MCSTV and image feature MSTM. It cannot only reflect the integrity and cooperativity of the action, but also express the spatial structure and temporal information more completely.

Let $\Gamma ={\left\{x_{i}^{1},x_{i}^{2},\cdots ,x_{i}^{M}\right\}}_{i=1}^{N}$ denote *N* action samples, and the *i*-th sample $\Gamma_{i}=\left\{x_{i}^{1},x_{i}^{2},\cdots ,x_{i}^{M}\right\}$ contains features from *M* different modalities, but they correspond to the same representation in a common space, denoted by $y_{i}$. Where $x_{i}$ is the sample of the *i*-th category, $y_{i}$ is the target projection center of the *i*-th category, modality *M* represents *M* types data.

In this paper, we propose Multi-Target Subspace Learning (MTSL) to study the common subspace of different features. The minimization problem is followed as:

(14) $$\begin{array}{c} \min_{U_{1},\cdots ,U_{M}}\sum_{p=1}^{M}{∥X_{p}^{T}U_{p}-Y∥}_{F}^{2}+\lambda_{1}\sum_{p=1}^{M}{∥U_{p}∥}_{21}+\lambda_{2}\sum_{p=1}^{M}\sum_{c=1}^{L-1}{∥X_{p}^{T}U_{p}-G_{c}∥}_{F}^{2} \end{array}$$

where $U_{p},p=1,2,\ldots ,M$ is the projection matrix of the *p*-th modality. $X_{p}$ is the sample features of the *p*-th modality before the projection. $X_{p}^{T}U_{p}$ is the sample features of the *p*-th modality after the projection. Y is the primary target projection matrix in the subspace, $Y={\left\{y_{1},y_{2},\cdots ,y_{N}\right\}}^{T}$. L is the number of categories. $\lambda_{1}$ and $\lambda_{2}$ are weighting parameters. $G_{c}$ is the *c*-th auxiliary projection target center matrix for samples of each category. The auxiliary projection target center is the symmetric target center of the projection target center of other categories with respect to the projection target center of the current category. The selections of $G_{c}$ are shown in Algorithm 1.
**Algorithm 1** The selection of $G_{c}$.**Input:** The target projection matrix of subspace: $Y={\left\{y_{1},y_{2},\cdots ,y_{N}\right\}}^{T}$; The number of categories: L.**Output:** The auxiliary projection target center matrix: $G_{c}$ A=Y  **for all**
c=1 to L−1
**do**   **for all**
j=1 to L
**do**    **if**
c==0
**then**     $B^{0}=A^{j-1}$    **else**     $B^{j}=A^{j-1}$    **end if**   **end for**   A=B   $G_{c}=2Y_{j}-A$  **end for****Note:**$B^{j}$ is the *j*-th column of *B*.

In Equation (14), the first term is used to learn the projection matrix, the second term is used for feature selection. The third item is used to expand the inter-class distance between different categories of samples and reduce the dimension of the projection target area.

According to the analysis of $l_{21}$-norm by He et al. [29], the second term is optimized. ${∥U_{p}∥}_{21}$ is deduced to $\mathrm{Tr}\left(U_{p}^{T}R_{p}U_{p}\right)$, where $R_{p}=\mathrm{Diag}\left(r_{p}\right)$, $r_{p}$ is an auxiliary vector of $l_{21}$-norm. The *i*-th parameter of $r_{p}$ is $r_{p}^{i}=\frac{1}{2{∥u_{p}^{i}∥}_{2}}$, $u_{p}^{i}$ is the *i*-th row vector of $U_{p}$. To keep the denominator from being 0, an infinite decimal α is introduced, and α is not 0. $r_{p}^{i}$ is rewritten as:

(15) $$\begin{array}{c} r_{p}^{i}=\frac{1}{2\sqrt{{∥u_{p}^{i}∥}_{2}^{2}+\alpha }} \end{array}$$

Equation (14) is derived and the computational formula of the projection matrix is obtained as:

(16) $$\begin{array}{cc} U_{p}= & {\left(X_{p}X_{p}^{T}+\lambda_{1}R_{p}+\lambda_{2}X_{p}X_{p}^{T}\right)}^{-1}\left(X_{p}Y+\lambda_{2}\sum_{c=1}^{L}X_{p}G_{c}\right) \end{array}$$

The projection matrix of different modalities is obtained through Equation (16), and the test sample of each modality are sent into the common subspace to acquire fusion features $X_{p}^{T}U_{p}$. The fusion features are used to research human action recognition.

## 4. Experiment

In this paper, the experiment runs on a desktop.The hardware is as follows: the main board is X370 Taichi, the CPU is 3.4 GHz R7 1700X, the graphics card is GTX 1660, and the memory is 16 GB. The software is Python 3.7 and Matlab 2018b. For each sequence, the average computing time of MCSTV is about 0.046 s, MSTM is about 1.239 s, and MSTM-HOG is about 0.043 s.

The experiments of proposed method were tested on Microsoft Research Action3D (MSR-Action3D) [5] and University of Texas at Dallas Multimodal Human Action Dataset (UTD-MHAD) [30]. Support vector machine (SVM) is used as a classifier to classify samples and obtain the final recognition accuracy. The dimension of skeleton feature MCSTV is related to the number of frames *N*, so we use Fisher Vector (FV) [31] to normalize MCSTV to make it linearly separable. After processing, the size of MCSTV is changed into 2pK×1. Where *p* is the number of rows of MCSTV, *K* is the clustering center of FV. In this paper, p=3, K=128, the size of MCSTV is 768×1.

### 4.1. Datasets and Experimental Settings

#### 4.1.1. MSR-Action3D

There are 557 depth sequences in this MSR-Action3D. The dataset includes 20 actions performed by 10 subjects, and each subject performs each action 2 to 3 times. The 20 actions are high wave, horizontal wave, hammer, hand catch, forward punch, high throw, draw x, draw tick, draw circle, hand clap, two hand wave, side boxing, bend, forward kick, side kick, jogging, tennis swing, tennis serve, golf swing, and pick up throw. The positive order actions of the dataset are denoted as D1. The positive order actions and reverse order actions of the dataset are denoted as D2. In this paper, two different settings are employed to this dataset.

Setting 1. Similar to Li et al. [5], D1 and D2 are divided into 3 groups (AS1, AS2, AS3). In addition, the MSR-Action3D subset dataset is shown in Table 1. The actions with high similarity are divided into the same group, in order to evaluate the model performance traits according to the training dataset size changes, each group of samples is tested to three experiments. In Test1, 1/3 samples are used as training data, and the remaining samples are used as test data. In Test2, 2/3 samples are used as training data, and the remaining samples are used as test data. In Test3, half subjects are used for training and the rest ones used for testing.

Setting 2. Similar to Chen et al. [20], all samples in the MSR-Action3D are classified at the same time. The samples of subject 1, 3, 5, 7, 9 are used as training data, and samples of subject 2, 4, 6, 8, 10 are used as test data.

#### 4.1.2. UTD-MHAD

There are 861 depth sequences in UTD-MHAD. The dataset includes 27 actions performed by 8 subjects (4 females and 4 males). Each subject performs each action 4 times. The 27 actions are swipe left, swipe right, wave, clap, throw, arm cross, basketball shoot, draw x, draw circle clockwise, draw circle counter clockwise, draw triangle, bowling, boxing, baseball swing, tennis swing, arm curl, tennis serve, push, knock, catch, pickup throw, jog, walk, sit2stand, stand2sit, lunge, squat. The positive order actions of the dataset are denoted as D3. The positive order actions and reverse order actions of the dataset are denoted as D4. In this paper, two different settings are employed to this dataset.

Setting 3. In order to evaluate the model performance traits according to the training dataset size changes, the samples in D3 and D4 are tested to three experiments. In Test1, 1/3 samples are used as training data, and the remaining samples are used as test data. In Test2, 2/3 samples are used as training data, and the remaining samples are used as test data. In Test3, we divid the samples into 5 parts and take turns to use 4 parts for training and 1 part for testing, the final result is the average of the 5 recognition rates.

Setting 4. Similar to Xu et al. [30], all samples in the UTD-MHAD are classified at the same time. The samples of subject 1, 3, 5, 7 are used to train, and samples of subject 2, 4, 6, 8 are used to test.

### 4.2. Parameter Selection

#### 4.2.1. Parameter Selection of ξ

**Figure 9:**
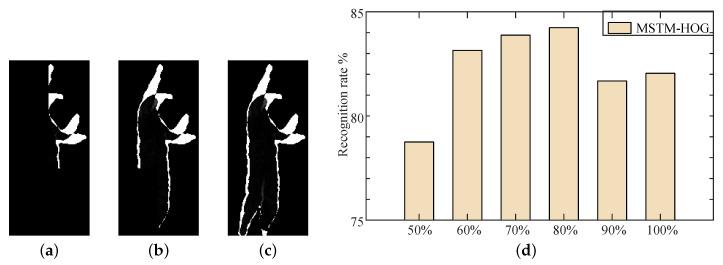
When setting different ξ, the effect of key motion energy retained. (**a**) ξ=50%; (**b**) ξ=80% (**c**) The original motion energy (**d**) The recognition result of MTSM-HOG when setting different ξ.

In the calculation of MSTM, we must use key information extraction based on inter-frame energy fluctuation algorithm to extract the key information in the motion energy. The amount of key information directly affects the ability of MSTM. Therefore, we need to set the appropriate ξ, which cannot only remove redundant information, but also retain key information completely. When setting different ξ, the effect of key motion energy retained is shown in Figure 9.

Figure 9a shows the key motion energy of tennis serve retained when ξ=50%. It can be seen that the value of ξ is too small, much key information is mistaken for redundant, and the possibility of this action being recognized as tennis serve is reduced. Figure 9b shows the key motion energy of tennis serve retained when ξ=80%. Compared with the original motion energy in Figure 9c, it not only retains the key information, but also removes the energy of the area that the motion is not obvious. Figure 9d shows the recognition rate of MTSM-HOG when setting different ξ according to Setting 2. It can be seen that MSTM-HOG achieves the highest recognition rate when ξ=80%. So we set ξ to 80% in the following experiment.

#### 4.2.2. Selection of Image Features

**Figure 10:**
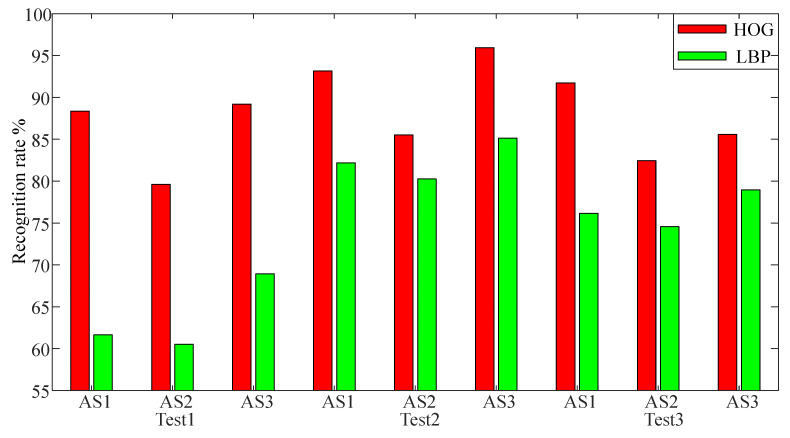
The recognition rate of MSTM when HOG features and LBP features are extracted.

Due to excessive noise, if the proposed MSTM is directly classified, the recognition results are affected. In this paper, we select Histogram of Oriented Gridient (HOG) [32] operator and Local Binary Pattern (LBP) [33] operator for feature extraction of image because they are not sensitive to light. HOG operator uses the image unit of 10×10 pixels to segment the image, combines every 2×2 image units into an image block, and slides the image block of 10 pixels to extract the HOG features of the image. LBP operator uses sampling radius of 2 and sampling point of 8 to extract the LBP features of the image. According to Setting 1, the results on D1 are shown in Figure 10 when HOG features and LBP features of MSTM are extracted.

As showed in Figure 10, the recognition accuracy of MSTM when extracting HOG features is higher than LBP. The LBP features mainly reflects the texture information around the pixel, while the HOG features can capture the image contour, and the main information of MSTM distributes in the image contour. Therefore, HOG features are more suitable for MSTM than LBP. In the following experiments, we only extract the HOG features of the images.

#### 4.2.3. Parameter Selection of $\lambda_{1}$ and $\lambda_{2}$

**Figure 11:**
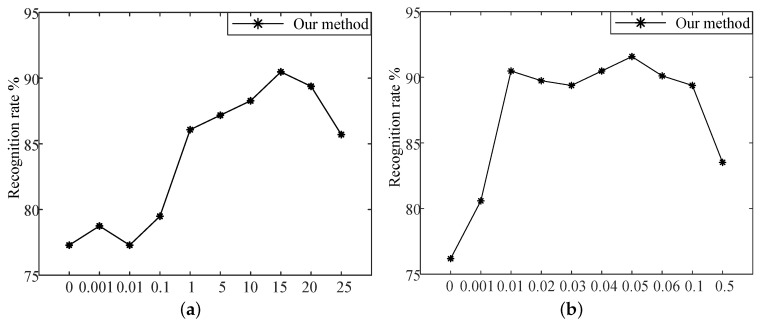
Parameter selection of $\lambda_{1}$ and $\lambda_{2}$. (**a**) Parameter selection of $\lambda_{1}$; (**b**) Parameter selection of $\lambda_{2}$.

When using MTSL to fuse image and skeleton features, we should select the fitting parameters $\lambda_{1}$ and $\lambda_{2}$. Assuming that $\lambda_{2}=0.01$, the optimal $\lambda_{1}$ is selected by enumerating different $\lambda_{1}$ and taking the recognition accuracy of our method as the evaluation standard. As can be seen from Figure 11a, when $\lambda_{1}=15$, our method achieves the optimal result. To obtain the optimal value of $\lambda_{2}$, we select the optimal value by enumerating different $\lambda_{2}$ when $\lambda_{1}=15$, and taking the recognition accuracy of our method as the evaluation criterion. As can be seen from Figure 11b, when $\lambda_{2}=0.05$, our method achieves the optimal result. In this experiment, the results are the recognition rate of our method on MSR-Action3D according to Setting 2.

### 4.3. Results and Discussion

#### 4.3.1. Evaluation of MCSTV

**Figure 12:**
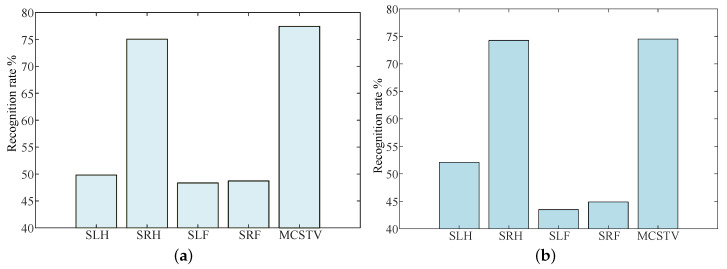
The recognition rate of different movement features. (**a**) The results on MSR-Action3D; (**b**) The results on UTD-MHAD.

MCSTV is a comprehensive vector formed by weighted accumulation of limb motion vectors. To verify that the accumulation of motion vectors can improve the action representation, we compare the recognition rate of each limb’s movement features with MCSTV. According to Setting 2, the results of different movement features on MSR-Action3D are shown in Figure 12a. According to Setting 4, the results on UTD-MHAD are shown in Figure 12b.

As shown in Figure 12a,b, MCSTV achieves the highest recognition rate on two datasets. Among them, it reaches 77.42% on MSR-Action3D and 74.5% on UTD-MHAD, both of them are higher than other movement features.

**Figure 13:**
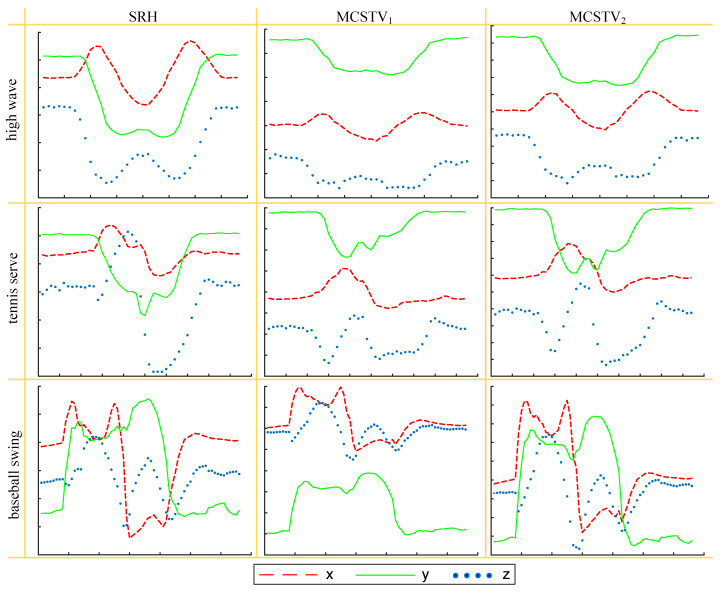
Various movement features of each action.

Next, we compare the variations of the three axes of various movement features. We select the actions with the right hand as the main movement limb and compares the expressive effect of SRH, ${\mathrm{MCSTV}}_{1}$ and ${\mathrm{MCSTV}}_{2}$. Where ${\mathrm{MCSTV}}_{1}$ is formed by the direct accumulation of each limb’s motion vectors, ${\mathrm{MCSTV}}_{2}$ is formed by the weighted accumulation of each limb’s motion vectors. We select high wave and tennis serve in MSR-Action3D, and select baseball swing in UTD-MHAD. Various movement features of each action are shown in Figure 13.

The main motion limb of the high wave is right upper limb, so the trajectory of MCSTV should be similar to SRH. Tennis serve and baseball swing are the actions that the movement of each limb is obvious, and right upper limb is the main motion joint, so the final MCSTV trajectory should be dominated by SRH. As shown in Figure 13, compared with ${\mathrm{MCSTV}}_{1}$, the trajectory of ${\mathrm{MCSTV}}_{2}$ is closer to SRH. In particular, the trajectory of baseball swing’s ${\mathrm{MCSTV}}_{2}$ is similar to SRH, but ${\mathrm{MCSTV}}_{1}$ is different. It can be explained that MCSTV formed by weighted accumulation is more accurate and highlight the main moving limbs.

**Figure 14:**
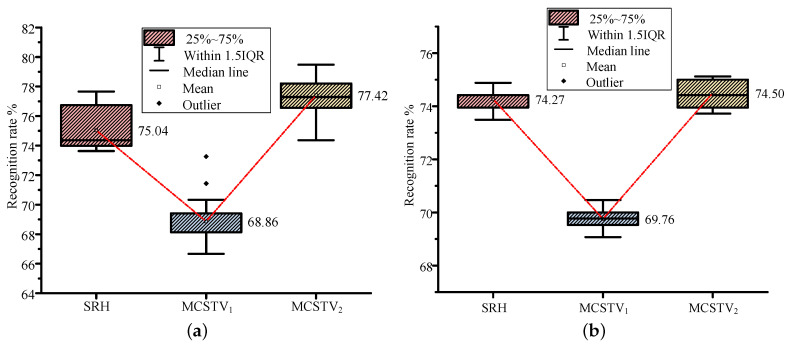
The recognition rate of SRH, ${\mathrm{MCSTV}}_{1}$ and ${\mathrm{MCSTV}}_{2}$. (**a**) The results on MSR-Action3D; (**b**) The results on UTD-MHAD.

Then, we verify that MCSTV formed by the weighted accumulation describes actions more accurately. The recognition rates are used as the criterion, ${\mathrm{MCSTV}}_{2}$ is compared with SRH and ${\mathrm{MCSTV}}_{1}$. The results on MSR-Action3D are shown in Figure 14a, and the results on UTD-MHAD are shown in Figure 14b.

The data in Figure 14a,b are the recognition rates of various features running 20 times. The figure clearly shows the mean, maximum, minimum, median, outlier, upper quartile, and lower quartile of the multiple results. It can be seen from the two figures that the recognition rate of ${\mathrm{MCSTV}}_{2}$ is higher than ${\mathrm{MCSTV}}_{1}$ and slightly higher than SRH. The main reason is that MCSTV formed by weighted accumulation not only considers the motion vector of each limb, but also gives different weight to each limb according to its contribution, which can highlight the information of the main moving limbs and describe actions more accurately.

#### 4.3.2. Evaluation of MSTM

MSTM expresses the change of motion energy with time on three orthogonal axes. It retains spatial structure and temporal information of actions. To verify that MSTM can completely retain the spatial structure of actions, the recognition rate of MSTM is compared with MEI, MHI and DMM when only positive order actions exist. In this experiment, according to Setting 1, the results of different methods on D1 are shown in Table 2. According to Setting 3, the results of different methods on D3 are shown in Table 3.

It can be seen from Table 2 and Table 3 that MSTM-HOG achieves or approaches the highest recognition accuracy in most tests. The reason is that MSTM represents the projection of motion energy on three orthogonal axes, which completely preserves the spatial structure of actions. By contrast, the recognition accuracy of MEI and MHI was lower in multiple tests. Due to MEI and MHI describe the largest contour of actions, there is coverage of front and behind motion information, MEI and MHI lose the motion information inside the contour. In addition, MEI and MHI do not involve the depth information of actions. DMM achieves the highest recognition accuracy in some tests, mainly it accumulates the motion energy from three views and completely retains the spatial information.

Next, we verify that MSTM has a strong ability to represent temporal information, the recognition rate of MSTM is compared with MEI, MHI and DMM when positive and reverse order actions exist. According to Setting 1, the results of different methods on D2 are shown in Table 4. According to Setting 3, the results of different methods on D4 are shown in Table 5.

In this paper, positive order high throw and reverse order high throw are considered to be two different actions. They have the same spatial trajectory and the opposite temporal information. So the number of actions in D2 is twice in D1, the number of actions in D4 is twice in D3. It can be known from Table 4 and Table 5 that the recognition rate of each method is lower than that of Table 2 and Table 3. MSTM maintains the highest recognition rate in all tests. The main reason is that MSTM splices the motion energy according to the temporal series, which can fully express the temporal information of actions. The MSTM of positive and reverse order actions are symmetric along the time axis, so the two actions can be accurately classified. By contrast, the recognition rate of MEI and DMM is lower in each test. The reason is that MEI and DMM could not express the temporal information. MEI and DMM of positive and reverse order actions are very similar and cannot be distinguished. MHI expresses part of the temporal information through brightness attenuation, so the recognition rate of MHI is higher than MEI and DMM, but far lower than MSTM.

#### 4.3.3. Evaluation of Feature Fusion

MCSTV can accurately describe the integrity and cooperativity of human limbs. MSTM can completely record the spatial structure and temporal information of actions. To combine the advantages of MSTM and MCSTV, we use MTSL to fuse MCSTV and MSTM-HOG. To prove that the fusion feature describes actions more accurately, we compare the recognition accuracy of the fusion algorithm with single algorithms. According to Setting 2, the results on MSR-Action3D are shown in Table 6. According to Setting 4, the results on UTD-MHAD are shown in Table 7.

It can be seen from Table 6 and Table 7 that the recognition accuracy of feature fused by MTSL algorithm is higher than single algorithms. The reason is that MTSL projects different features into a common subspace and complements the advantages of each single features.

It can be also known that the recognition accuracy of feature fused by MTSL is higher than CCA and DCA. Mainly, the MTSL algorithm constructs multiple projection targets to make the subspace samples converge to the hyperplane near the multiple projection target centers and increases the distance between the subspace samples. However, CCA and DCA mainly describe the correlation of two features, image and skeleton are two different modals with small correlation.

### 4.4. Comparison with Other Methods

We compare our method with other methods. According to Setting 2, the recognition accuracy comparison with other methods on MSR-Action3D is shown in Table 8, According to Setting 4, the recognition accuracy comparison with other methods on UTD-MHAD is shown in Table 9.

It can be seen from Table 8 and Table 9 that the recognition accuracy of our method reached 91.58% on the MSR-Action3D and 89.53% on the UTD-MHAD, both of which were higher than the recognition accuracy of other methods listed. The evaluation results indicate the superiority of our method.

## 5. Conclusions

In this paper, we propose an action feature representation that considers the integrally cooperative movement features of human action, called MCSTV and MSTM. MCSTV accumulates weighted limbs’ motion vector to form a new vector and uses this vector to account for the movement features of actions. MSTM algorithm projects key motion energy that extracted by key information extraction based on inter-frame energy fluctuation to three orthogonal axes and stitches them in temporal series to construct the MSTM. To describe the action information more accurately, the MTSL algorithm is used to fuse MCSTV and MSTM-HOG. The experimental results on MSR-Action3D and UTD-MHAD show that MCSTV not only considers the integral and cooperative between the motion joints, but also highlights the main moving limbs of the body. Compared with MEI, MHI, and DMM, MSTM describes the spatial structure and temporal information better. The recognition accuracy of features fused by MTSL algorithm is higher than most existing algorithms.

## 6. Future Expectations

When we use key information extraction based on inter-frame energy fluctuation algorithm to extract the key information, in some cases, the redundancy cannot be effectively removed because the habitual shaking of some joints is too sharp. Next, we will focus on how to effectively remove the redundant information.

## Figures and Tables

**Table 1:** MSR-Action3D subset dataset

	Action Set 1 (AS1)	Action Set 2 (AS2)	Action Set 3 (AS3)
Action name	horizontal wave	high wave	high throw
	hammer	hand catch	forward kick
	forward punch	draw x	side kick
	high throw	draw tick	jogging
	hand clap	draw circle	tennis swing
	bend	two hand wave	tennis serve
	tennis serve	forward kick	golf swing
	pick up throw	side boxing	pick up throw

**Table 2:** The recognition rates (%) of different methods on D1.

Method	Test1				Test2				Test3			
	AS1	AS2	AS3	Average	AS1	AS2	AS3	Average	AS1	AS2	AS3	Average
MEI-HOG	73.29	73.03	72.30	72.87	86.30	86.84	90.54	87.89	86.24	81.58	71.17	79.66
MHI-HOG	69.86	64.47	72.97	69.10	86.30	88.16	90.54	88.33	83.49	81.58	72.07	79.05
DMM-HOG	76.03	71.71	77.70	75.15	87.67	86.84	94.59	89.10	84.40	85.09	75.68	81.72
MSTM-HOG	88.36	79.61	89.19	85.72	93.15	85.53	95.95	91.54	91.74	82.46	85.59	86.60

**Table 3:** The recognition rates (%) of different methods on D3.

Method	Test1	Test2	Test3
MEI-HOG	69.51	83.28	89.53
MHI-HOG	72.47	89.55	94.19
DMM-HOG	78.57	94.08	98.84
MSTM-HOG	85.89	94.77	96.51

**Table 4:** The recognition rates (%) of different methods on D2.

Method	Test1				Test2				Test3			
	AS1	AS2	AS3	Average	AS1	AS2	AS3	Average	AS1	AS2	AS3	Average
MEI-HOG	29.11	33.88	29.05	30.68	30.14	25.66	33.78	29.86	36.99	35.53	32.43	34.98
MHI-HOG	37.67	39.80	36.49	37.97	40.41	48.68	45.27	44.79	44.75	48.25	38.29	43.76
DMM-HOG	34.93	34.87	27.70	32.50	32.19	32.24	31.08	31.84	38.36	35.52	30.63	34.84
MSTM-HOG	80.48	69.74	84.46	78.22	83.56	83.55	91.89	86.33	85.84	81.58	90.54	85.99

**Table 5:** The recognition rates (%) of different methods on D4.

Method	Test1	Test2	Test3
MEI-HOG	28.66	33.97	32.85
MHI-HOG	44.60	60.63	66.86
DMM-HOG	38.85	39.72	35.47
MSTM-HOG	83.80	92.68	94.48

**Table 6:** The results on MSR-Action3D, according to Setting 2.

Method	Recognition Rate (%)
MEI-HOG	69.23
MHI-HOG	69.96
DMM-HOG	85.35
MSTM-HOG	84.24
MCSTV	77.42
CCA + MCSTV + MSTM-HOG	67.77
DCA + MCSTV + MSTM-HOG	75.09
Our Method	91.58

**Table 7:** The results on UTD-MHAD, according to Setting 2.

Method	Recognition Rate (%)
MEI-HOG	49.77
MHI-HOG	57.21
DMM-HOG	79.07
MSTM-HOG	83.72
MCSTV	74.50
CCA + MCSTV + MSTM-HOG	63.95
DCA + MCSTV + MSTM-HOG	77.21
Our Method	89.53

**Table 8:** The recognition accuracy comparison with other methods on MSR-Action3D.

Method	Recognition Rate (%)
DMM-HOG [7]	85.5
HON4D [6]	88.89
Bag of 3D points [5]	74.7
DMM-GLAC-FF [21]	89.38
Random Occupancy Patterns [23]	86.5
Depth Cuboid [24]	89.3
STOP [25]	84.8
Our Method	91.58

**Table 9:** The recognition accuracy comparison with other methods on UTD-MHAD.

Method	Recognition Rate (%)
Kinect [30]	66.1
Kinect + Inertial [30]	79.1
3DHoT-MBC [22]	84.4
Our Method	89.53
